# Comparison of Perinatal Outcomes Following Elective and Emergency Cerclage Insertion: A Ten-Year Retrospective Cohort Study

**DOI:** 10.3390/jcm14103515

**Published:** 2025-05-17

**Authors:** Franciszek Ługowski, Julia Babińska, Kamil Jasak, Karolina Pastwa, Ewelina Litwińska-Korcz, Magdalena Litwińska, Zoulikha Jabiry-Zieniewicz, Monika Szpotańska-Sikorska

**Affiliations:** 1st Department of Obstetrics and Gynecology, Medical University of Warsaw, 02-015 Warsaw, Poland

**Keywords:** cervical cerclage, elective cerclage, emergency cerclage, cervical insufficiency, preterm birth

## Abstract

**Background**: Cervical insufficiency (CI) is a painless cervix dilation in the second or early third trimester due to a structural or functional defect. However, CI is often diagnosed retrospectively. A cervix with CI cannot retain the fetus. This condition significantly increases the morbidity associated with extreme prematurity. Women diagnosed with cervical incompetence and dilatation in the mid-second trimester are offered interventions to prolong the duration of pregnancy, with the mainstay of therapy being emergency cerclage. A prophylactic cerclage may be offered to women with a history of extremely preterm birth due to isthmic cervical incompetence. **Aim**: The aim of this study was to evaluate the perinatal outcomes of elective and emergency cerclages. **Materials and Methods**: A 10-year retrospective analysis, from 1 January 2015 to 29 February 2024 of pregnancies with indications for cervical cerclage, was conducted. Obstetric and neonatal outcomes were assessed. **Results**: Prophylactic cervical cerclage was performed in 43 (57%) and emergency cerclage in 32 (43%) of all analyzed cases. The mean prolongation of gestation (measured as the period between cerclage insertion and delivery) was higher in the elective cerclage group compared with the emergency cerclage group (18.6 ± 5.4 vs. 12.2 ± 6.4 weeks; *p* < 0.0001). The mean gestational week at cerclage removal was also higher in the elective group (36.1 ± 2.2 vs. 31.4 ± 5.6 weeks; *p* < 0.001). Deliveries in the extreme prematurity period (before 28 completed weeks of gestation) were five times more often in the rescue cerclage group. A significantly higher mean birthweight was reported in the elective cerclage group, at 2920.4 ± 946.8 g vs. 2078.8 ± 1147.8 g (*p* = 0.0004). Emergency cerclage insertion was associated with a higher need for NICU hospitalization (28% vs. 5%, *p* = 0.003), continuous positive airway pressure (38% vs. 2%, *p* < 0.0001), and intubation (22% vs. 0%, *p* = 0.003). **Conclusions**: While elective cerclage is associated with more favorable perinatal and neonatal outcomes, this likely reflects earlier intervention in lower-risk pregnancies rather than inherent superiority of the approach. Emergency cerclage, performed under urgent and often suboptimal conditions, remains a critical and effective intervention capable of prolonging gestation and improving neonatal survival in high-risk cases.

## 1. Introduction

Cervical insufficiency (CI) is a painless cervical dilation in the second or early third trimester due to a structural or functional defect [1]. However, CI is often diagnosed retrospectively. A cervix with CI cannot retain the fetus [2]. The condition may lead to preterm premature rupture of membranes (PPROM), pregnancy loss, or preterm birth (PTB) [1,3]. Because CI occurs in approximately 1% of the obstetric population and 8% of women with recurrent mid-second trimester losses, it constitutes a frequent and pivotal issue [1]. Previous PPROM, miscarriage, a shortened cervix, uterine abnormalities, and cervical trauma are significant risk factors for CI [1]. In addition, numerous studies have also identified an association between polycystic ovarian syndrome (PCOS) and CI [4]. The etiology of CI is multifaceted; both congenital and acquired defects of the cervix can be causative. However, congenital impairments, such as Müllerian duct anomalies, deficient collagen production, and in utero exposure to diethylstilbestrol, are less common [5]. More frequently, CI is caused by cervical trauma, which may be a result of cervical lacerations at delivery, cervical conization, or loop electrosurgical excision procedures [6]. Moreover, unintentional incision into the uterine cervix during a cesarean section or other procedure, such as hysteroscopy, may also provoke CI in future pregnancies [7].

Currently, there are no diagnostic tests for CI, so the diagnosis is partly based on the exclusion of other causes of preterm delivery or mid-second trimester pregnancy loss [1,8]. However, the use of transvaginal ultrasonography has become more frequent in the assessment of the cervix, as cervical shortening below 25 mm significantly increases the risk of preterm delivery [9,10]. The management options for women with a short cervix or CI include vaginal progesterone, cervical pessaries, and cervical cerclages. Cervical cerclages are a significant management option in pregnancies complicated with CI [11].

Cervical cerclages strengthen the cervix, allowing it to retain length and preserve the mucus plug at the cervical opening in order to protect against ascending infections [12]. The two main techniques of transvaginal cerclage are the McDonald approach, in which the suture is inserted close to the junction of the cervix with the vagina, and the Shirodkar approach, which involves the insertion of the suture above the junction [13]. Both methods have comparable efficacy and are well established to decrease the rate of second-trimester miscarriage and preterm delivery [14]. In this study, we analyzed the perinatal outcomes of elective and emergency cerclages.

According to the American College of Obstetricians and Gynecologists (ACOG) recommendations, indications for cerclage placement should be based on a history of CI, obstetric examination findings in the current pregnancy, or a history of preterm birth and ultrasonographic findings in the current pregnancy. Indications based solely on history are so-called prophylactic (elective) cerclages. There are also emergency (rescue) cerclages with the indications based on physical examination, also taking the woman’s history into consideration [3]. Notably, emergency cerclages are performed under more acute and challenging clinical conditions—often with advanced cervical dilation, exposed membranes, or signs of imminent pregnancy loss. As such, they inherently differ from prophylactic cerclages in timing and clinical context, making direct comparisons between the two groups inappropriate without accounting for these baseline disparities.

The presented study aimed to evaluate the perinatal outcomes of emergency and elective cervical cerclages in a tertiary referral center.

## 2. Materials and Methods

The study was retrospectively designed and included pregnant women who underwent cervical cerclage between 1 January 2015 and 29 February 2024 and afterward delivered at the 1st Department of Obstetrics and Gynecology of the Medical University of Warsaw. The study protocol was reviewed and approved on 16 December 2024 by the Ethics and Research Committee of the Medical University of Warsaw (approval no. AKBE/344/2024). The patients’ information, such as age, number of pregnancies, number of deliveries, history of CI, history of cervical cerclage, maternal white blood count (WBC), C-reactive protein (CRP) and procalcitonin (PCT) levels, gestational week at the moment of cervical stitch and delivery, as well as newborn well-being, were obtained from the patient records. Neonatal data, such as Apgar score, birthweight, neonatal intensive care unit (NICU) hospitalization, the necessity of the use of mechanical ventilation, and continuous positive airway pressure, were also evaluated.

In order to maintain the homogeneity of the study population, only single pregnancies were included in the final analysis, thus excluding multiple pregnancies. Other exclusion criteria included delivery in a center other than the one where the cervical cerclage procedure was performed. This decision resulted from the retrospective analysis and, thus, the impossibility of obtaining complete perinatal and neonatal data. Women with systemic or gestational complications, such as gestational hypertension or diabetes, were not excluded from the study, as these conditions were not considered to directly influence the outcomes related to cervical cerclage efficacy. However, their presence was not analyzed as an independent variable in this study.

All eligible women were meticulously informed about the possible advantages and disadvantages of conservative management and cervical cerclage for asymptomatic cervical shortening or dilation. The included patients were then categorized into the elective cerclage and emergency cerclage groups.

Experienced obstetricians at our tertiary institution performed both singular and double cerclages using the McDonald, Wurm–Hefner, and Hervet techniques.

Women with CI were qualified for the procedure of elective cervical cerclage insertion based on the following criteria: a history of second-trimester pregnancy loss related to painless cervical dilation, or a history of cerclage insertion in a previous pregnancy, with placement typically between 12–14 weeks of gestation. This timing aligns with ACOG and RCOG recommendations for history-indicated cerclage [3,13]. Cervical length measurement was not used as a primary criterion in this group at that gestational age.

Emergency cerclage was performed in pregnant women who presented with painless cervical dilation with a protruding amniotic sac before 26 weeks of gestation [15]. While many of these women also had a history suggestive of cervical insufficiency, they were not identified or referred early enough for elective cerclage due to late presentation, limited prior documentation, or unclear risk history. In such cases, the decision for cerclage was made based on acute clinical findings rather than historical risk alone.

This study included both elective and emergency cerclage cases to comprehensively assess outcomes across the clinical spectrum of cervical insufficiency. These two approaches are inherently applied in markedly different scenarios: elective cerclages are placed early in asymptomatic patients based on history or ultrasound findings, whereas emergency cerclages are performed later under urgent conditions, often involving cervical dilation or bulging membranes. Including both groups aligns with existing research, as it enables a meaningful evaluation of perinatal outcomes relative to the clinical context and timing of intervention, rather than a direct comparison between inherently unequal populations.

In selected cases, a cervical pessary was inserted as an adjunct to cerclage during follow-up, typically in the second or early third trimester, due to ongoing cervical shortening or signs of cervical funneling despite the presence of a cerclage. This intervention was based on individualized clinical judgment and was not applied at the time of cerclage insertion. The cervical pessary was used as a secondary, supportive measure in selected patients who demonstrated progressive cervical shortening or funneling after cerclage placement. Given that pessary insertion was based on dynamic clinical need and was equally distributed between groups (24/43 in the elective group and 18/32 in the emergency group), it is unlikely to have introduced systematic bias between the two arms of the study.

All patients received standard supportive care following cerclage placement. Vaginal micronized progesterone (200 mg daily) was administered postoperatively in selected patients from both groups and continued until 36 weeks of gestation or delivery. Routine tocolysis was not employed; however, magnesium sulfate was administered for fetal neuroprotection in selected cases at risk of preterm birth before 32 weeks of gestation, as per institutional protocol.

Antenatal corticosteroids (two doses of 12 mg betamethasone intramuscularly, 24 h apart) were administered to women at risk of imminent preterm delivery between 24 + 0 and 34 + 0 weeks of gestation, in accordance with national obstetric guidelines.

Empirical antibiotic therapy was administered perioperatively in patients undergoing cervical cerclage, especially in emergency cases with a protruding amniotic sac or suspected subclinical infection. Antibiotic choice and duration were determined by treating clinicians and institutional practices. In cases of a protruding amniotic sac or visible sludge on ultrasound, the antibiotic regimen, including ceftriaxone 1 g (intravenous) every 24 h, clarithromycin 500 mg (oral) every 12 h, and metronidazole 500 mg (intravenous) every 8 h, was administered for 7 days [16]. Due to the retrospective nature of the study, collecting full detailed data from 10 years ago regarding the time, dosage and all antibiotic regimens used proved difficult to implement. The use of incomplete data could distort the analysis of the obtained results. 

The primary outcomes included the week of delivery, birthweight, and pregnancy prolongation following cervical cerclage insertion. Secondary outcomes comprised neonatal mortality, need for NICU hospitalization, Apgar score, birthweight, as well as maternal WBC and CRP levels.

The highest maternal white blood cell count (WBC) and C-reactive protein (CRP) values during hospitalization for cerclage insertion were recorded and analyzed as surrogate markers of inflammation or subclinical infection. Serial measurements or microbiological data throughout the pregnancy were not consistently available and were therefore not included in the analysis.

Congenital infections were defined as clinical or laboratory evidence of neonatal infection diagnosed within the first 72 h of life, likely acquired in utero. These included early-onset neonatal sepsis based on clinical symptoms (e.g., respiratory distress, temperature instability) and elevated inflammatory markers. Due to the retrospective design, data on specific pathogens or culture-confirmed infections were limited.

The data were entered using Microsoft Office Excel software version 14.0 and analyzed by using the Statistical Package for Social Sciences (SPSS) 20.0 (Armonk, NY, USA). Data are expressed as mean  ±  standard deviation. Chi-square (χ^2^) tests of independence were used for the analysis of categorical variables. For non-categorical variables, normally distributed data were analyzed with Student’s *t*-test, while non-normally distributed data were analyzed with the Mann–Whitney *U* test. All *p*-values were considered statistically significant if below 0.05.

## 3. Results

Between 1 January 2015 and 29 February, 114 women were diagnosed with CI in our department and underwent the procedure of cervical cerclage insertion, of whom 39 were excluded from further analysis—34 due to incomplete perinatal data and 5 due to twin pregnancies, leaving a total of 75 patients with singleton pregnancies. Elective cerclages were performed in 43 (57%) pregnant women, whereas emergency cerclages were performed in 32 patients (43%). A pessary was inserted in 42 patients with CI. In the elective cerclage group, there was a single miscarriage, while in the emergency group, two miscarriages and two stillbirths were noted. The mean age of patients enrolled in the study was 32.7 (range 20–42 SD ± 5.2), the mean number of pregnancies was 3.09 (range 1–9 SD ± 1.8), and the mean number of deliveries was 1.8 (range 1–5 SD ± 1.0). Patients’ mean gestational age at diagnosis was 18.7 weeks of gestation (range 12–31 SD ± 3.7) and mean gestational age at the time of the procedure was 18.8 weeks of gestation (range 12–31 SD ± 3.6). The mean gestational age at delivery after the cerclage insertion was 34.7 weeks (range 24–41 SD ± 4.9), and the mean time between the procedure and delivery was 16.0 (range 1–26 SD ± 6.8) weeks. A cervical pessary was inserted in 42 patients following cerclage placement due to concerns of progressive cervical shortening. This included 24 patients in the elective cerclage group and 18 in the emergency cerclage group. Due to heterogeneity in indication and timing, we did not assess the pessary + cerclage subgroup as a separate analytical cohort.

The characteristics of elective and emergency cerclage groups are assessed in Table 1. The mean gestational age at diagnosis was significantly lower in the elective group (17.5 ± 3.5 vs. 20.2 ± 2.6, weeks *p* < 0.0001), similar to the mean gestational age at the time of the procedure (17.5 ± 3.5 vs. 20.2 ± 2.6, weeks *p* < 0.0001). Moreover, we observed a higher mean maternal age in the elective cerclage group (32.6 ± 5.1 years of age) in comparison with the emergency cerclage group (31.9 ± 5.8 years of age), but the difference was not statistically significant (*p* = 0.27). In our study, we also evaluated the mean number of pregnancies (including the current one) among the enrolled women, which was greater in the elective cerclage group (3.3 ± 2.1 vs. 2.4 ± 1.7, *p* = 0.007). Likewise, the number of deliveries (including the current one) was higher among the elective cerclage patients compared to the emergency cerclage group (1.9 ± 1.1 vs. 1.4 ± 0.8, *p* = 0.013).

**Figure 1:**
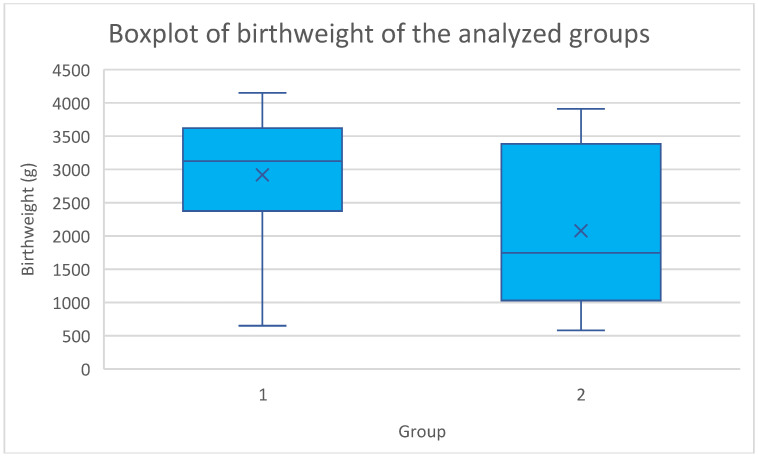
A boxplot illustrating the distribution of birthweight across the elective and emergency cerclage groups. 1—elective cerclage group; 2—emergency cerclage group.

In addition, we assessed the neonatal outcomes in the analyzed obstetric population (Table 2). Our study revealed that the mean birthweight and mean Apgar scores at 1, 5, and 10 min were statistically higher in the elective cerclage group. The distribution of birthweight in the analyzed groups is illustrated in Figure 1. On the other hand, the need for NICU hospitalization, CPAP, and intubation, as well as congenital infections, occurred more frequently in the emergency cerclage group. All of the above differences were statistically significant. Also, we recorded more stillbirths and miscarriages in the emergency cerclage group but the differences were not statistically significant.

**Figure 2:**
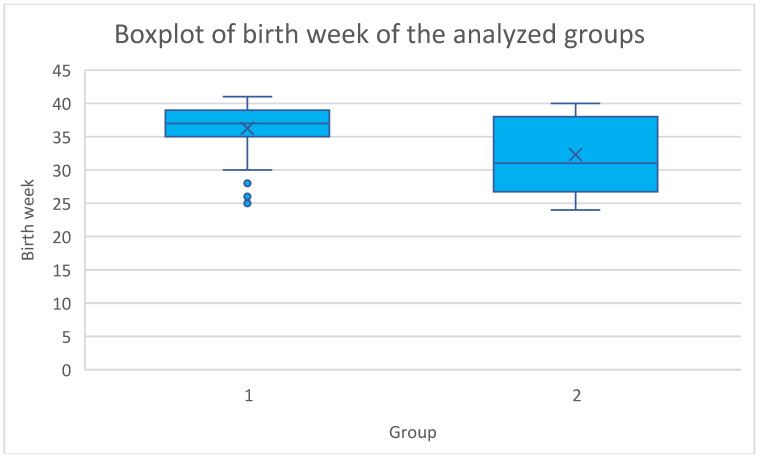
A boxplot illustrating the distribution of the birth week across the elective and emergency cerclage groups. 1—elective cerclage group; 2—emergency cerclage group.

Thirdly, our study aimed to analyze the perinatal and maternal outcomes following cervical cerclage insertion (Table 3). We noted a significant difference between the groups regarding the mean gestational age at delivery—36.1 ± 3.88 and 31.4 ± 5.68 weeks of gestation, *p* < 0.001—in the elective and emergency groups, respectively. The distribution of birth week in the analyzed groups is illustrated in Figure 2. Regarding the mode of delivery, cesarean section was performed in 23 (53%) patients from the elective cerclage group and 19 (59%) from the emergency cerclage group (*p* = 0.172). PPROM occurred in six (14%) pregnant women in the elective cerclage group and four (13%) patients who underwent emergency cerclage; however, the difference was not statistically significant (*p* = 0.416). We found that elective cerclages were positively associated with delivery after 38 weeks of gestation (*p* = 0.041). Contrary to that, emergency cerclages correlated with delivery before 28 weeks of gestation (*p* < 0.0001). No statistically significant differences were found for delivery at 28–32 and 33–38 gestational weeks. Importantly, the mean prolongation of pregnancy (measured as the period between the insertion of cervical cerclage and labor) was higher in the elective cerclage group compared to the emergency cerclage group (18.6 ± 5.4 vs. 12.2 ± 6.4 weeks, *p* < 0.0001). The mean gestational week at cerclage removal was also higher in the elective group (36.1 ± 2.2 vs. 31.4 ± 5.6 weeks, *p* < 0.001). Regarding maternal outcomes, the mean white blood count (WBC) was statistically greater in the emergency group. No significant difference between the groups was observed in the maternal C-reactive protein (CRP) levels. In addition, a lack of statistical significance of the differences was noted regarding the need for pre-birth MgSO4 administration and cervical pessary insertion.

## 4. Discussion

The management options for women with a short cervix or CI include vaginal progesterone, cervical pessaries, and cervical cerclages [11]. Vaginal progesterone has been proven to reduce the rate of PTB and neonatal morbidity and is recommended for women with no prior spontaneous PTB with a cervical length below 25 mm until 36 weeks of gestation [17,18]. The support for cervical pessary use in the management of CI has been ambiguous. Some studies suggested efficacy comparable to progesterone treatment [19,20], while others revealed no or little decrease in the rate of PTB [21].

Our analysis revealed a significant difference between the elective cerclage and emergency groups regarding GW at the time of the procedure, GW at the time of diagnosis, number of pregnancies, and number of deliveries. Numerous publications revealed a lower GW at the time of CI diagnosis and suture insertion in the elective cerclage group, similar to our analysis [22,23,24]. In addition, we found no statistically significant differences regarding maternal age, which has been confirmed in previous studies [22,23].

Moreover, our comparison of neonatal outcomes between the elective cerclage and emergency cerclage groups showed a significantly lower birthweight in the emergency group, as well as a more frequent need for NICU hospitalization, CPAP, and intubation. In our study, we noted a significant difference regarding mean birthweight between the elective and emergency cerclage groups—2920.4 ± 946.8 g and 2078.8 ± 1147.8 g, respectively (*p* = 0.0004). In interpreting neonatal outcomes, it is essential to recognize the fundamentally different clinical contexts of elective and emergency cerclage. Elective cerclage is typically performed in a stable setting during the early second trimester, allowing for a greater window to prevent preterm birth and optimize neonatal outcomes. In contrast, emergency cerclage is a reactive measure, often applied in acute situations with advanced cervical changes and a higher baseline risk for extreme prematurity, infection, and neonatal morbidity. Numerous studies have consistently reported significantly higher mean birthweights in elective cerclage groups compared to emergency cerclage populations [22,25,26]. Moreover, Jafarzade et al. also reported a higher need for NICU hospitalization and intubation in the emergency cerclage group [22]. Unlike the above-mentioned studies, we also assessed the need for CPAP, which turned out significantly higher in the emergency cerclage group (12 vs. 1 event, *p* < 0.0001). Furthermore, in our study, stillbirths and miscarriages occurred more frequently in the emergency group, which is consistent with previous publications [22,26]. Of importance, we also recorded a higher mean Apgar score at 1, 5, and 10 min in the elective cerclage group, along with a lower incidence of congenital infections. The mean Apgar at 1 min was higher in the elective cerclage group in a study by Liddiard et al.; however, the difference was not significant (seven vs. four, *p* = 0.28) [23]. In addition, a retrospective analysis conducted in India revealed higher Apgar scores at 1 and 5 min in the elective compared to the emergency cerclage group, but both differences were not significant [25]. However, the presence of a higher 1 min Apgar score in the elective cerclage group was also confirmed in a meta-analysis by Wei et al. (WMD = 2.8720, 95% CI 2.105–3.639, *p* = 0.000) [27]. Cervical cerclages do not increase the risk of infections; however, CI itself is associated with an increased rate of intrauterine infections [27,28]. Moreover, the incidence of infections further rises in acute CI cases by up to 80% [27,29,30]. In our study, we recorded a higher infection rate in the emergency cerclage group, which is consistent with the aforementioned studies. Of importance, the use of antibiotics in CI has not been found to affect the Apgar score [27]. While our study found significantly better neonatal outcomes—including higher birthweight, Apgar scores, and lower rates of NICU admission, CPAP, and intubation—in the elective group, these differences must be interpreted in light of the underlying risk profiles. Rather than reflecting the superiority of one approach, these findings highlight the value of timely intervention and underscore the remarkable ability of emergency cerclage to stabilize pregnancies despite high-risk presentations.

In our study, we noted a mean birth week of 36.1 ± 3.88 of gestational age in the elective cerclage group and 31.4 ± 5.68 in the emergency cerclage group (*p* < 0.001). Moreover, the outcomes of our analysis indicate that the emergency cerclage group was significantly associated with delivery before 28 weeks of gestation, as well as between 28 and 32 weeks of gestation. In addition, the elective cerclage group correlated with delivery after 38 weeks of gestation. Maternal outcomes in our study also reflect the differing clinical scenarios between elective and emergency cerclage. The significantly higher maternal white blood cell (WBC) count observed in the emergency group may indicate a greater inflammatory burden or subclinical infection, which is consistent with the more advanced cervical changes and urgency at presentation in this group. Importantly, no significant differences were noted in C-reactive protein (CRP) levels or the incidence of PPROM between the two groups. Comparable results have been observed by Lidiard et al., who reported a higher mean GW at delivery in the elective cerclage group (32 vs. 26, *p* = 0.24) [23]. Moreover, the same association was depicted by Jafarzade et al., and in that study, the difference was statistically significant (34.6 vs. 30.8, *p* = 0.000) [22], consistent with Kumari et al. (34.2 vs. 32.2, *p* = 0.13) [31], Vasuveda et al. (37.0 vs. 34.0) [26] and Khan et al. (36.0 vs. 32.4) [25]. On the other hand, in a retrospective study conducted in Israel, there was a higher rate of births between 24 and 28 GW in the emergency cerclage group; however, the difference was not statistically significant [32]. Moreover, no significant differences regarding GW at delivery between the elective and emergency cerclage groups were found [32]. Of importance, we recorded a significantly longer prolongation of pregnancy following cerclage insertion in the elective group—18.6 ± 5.4 vs. 12.2 ± 6.4 weeks, *p* < 0.0001. These results are consistent with previous literature data. For instance, an Australian retrospective cohort study described a prolongation of 21.4 weeks in the elective cerclage group compared to 14.1 weeks in the emergency cerclage group [26]. The same tendency was noted in an Indian population with similar outcomes—22.28 ± 5.06 weeks in the elective cerclage group and 11.65 ± 8.16 weeks in the emergency cerclage one [25]. The above outcomes suggest the importance of an early diagnosis of CI and the numerous advantages of elective cerclages regarding the prolongation of pregnancy and, hence, positive perinatal outcomes. These findings further support the notion that emergency cerclages are performed in more challenging conditions, and while maternal inflammation markers may be elevated, the procedure itself remains a viable and safe option for prolonging pregnancy even in high-risk contexts.

Although the elective cerclage group demonstrated more favorable perinatal outcomes overall, it is important to emphasize that the emergency cerclage group included patients with significantly more advanced cervical changes and higher baseline risk. The ability of emergency cerclage to prolong pregnancy and improve neonatal survival in such critical scenarios underscores its value as an essential intervention in modern obstetric practice. These findings support the need for both proactive identification of at-risk pregnancies and the availability of timely emergency cerclage when indicated.

One of the limitations of our study is the lack of reliable data on the incidence of chorioamnionitis. Furthermore, the actual assessment of chorioamnionitis with the use of an amniocentesis procedure and amniotic fluid testing was not permanently implemented until a few years ago; hence, the actual assessment of the presence of chorioamnionitis would be rather objective. Due to inconsistent documentation and the retrospective design of this study, we could not include this outcome in our analysis. Prospective studies with standardized infection surveillance protocols are needed to better assess this risk. We acknowledge that the use of an adjunctive pessary in over half the cohort may have influenced outcomes and represents a limitation of the retrospective design. Future prospective studies are needed to evaluate the independent and combined effects of cerclage and pessary in the management of cervical insufficiency. Moreover, the relatively low rate of magnesium sulfate administration for fetal neuroprotection in eligible cases (<32 weeks gestation) may be attributed to variability in clinical practice over the 10-year study period and differences in the timing or perceived imminence of preterm delivery. This highlights the need for more consistent implementation of standardized neuroprotective protocols. Furthermore, due to the retrospective design, data regarding previous modes of delivery (e.g., prior cesarean section) were not consistently documented and thus could not be analyzed. Additionally, while we now report mode of delivery outcomes in the current pregnancy, their interpretation should be made cautiously, given potential confounders.

## 5. Conclusions

Emergency cerclage effectively prolongs pregnancy and is a safe procedure in urgent cases. As they are performed in urgent cases under difficult circumstances, it must be noted that they provide very good clinical outcomes that cannot be directly compared with elective cerclages. While elective cerclage is associated with more favorable perinatal and neonatal outcomes, this likely reflects earlier intervention in lower-risk pregnancies rather than any inherent superiority of the approach. Emergency cerclage, performed under urgent and often suboptimal conditions, remains a critical and effective intervention capable of prolonging gestation and improving neonatal survival in high-risk cases. These findings underscore the importance of both timely identification of at-risk pregnancies for prophylactic intervention and the availability of rescue cerclage as a life- and pregnancy-saving measure in advanced cervical insufficiency.

## Figures and Tables

**Table 1:** The demographic characteristics of the study population.

	Elective (n = 43)	Emergency (n = 32)	*p*
Maternal age (mean), years	32.6 ± 5.1	31.9 ± 5.8	0.27
History of cervical insufficiency	27 (63%)	24 (75%)	0.446
GW in procedure (mean)	17.5 ± 3.5	20.2 ± 2.6	<0.0001
GW week at diagnosis (mean)	17.5 ± 3.5	20.2 ± 2.6	<0.0001
Number of pregnancies (mean)	3.3 ± 2.1	2.4 ± 1.7	0.007
Number of deliveries (mean)	1.9 ± 1.1	1.4 ± 0.8	0.013
Progesterone administration	36 (84%)	26 (81%)	0.85

GW—gestational week.

**Table 2:** Comparison of neonatal outcomes between patients with elective and emergency cerclages.

	Elective (n = 43)	Emergency (n = 32)	*p*
Birthweight (mean), g	2920.4 ± 946.8	2078.8 ± 1147.8	0.0004
Need for NICU hospitalization	2 (5%)	9 (28%)	0.003
Need for CPAP	1 (2%)	12 (38%)	<0.0001
Need for intubation	0 (0%)	7 (22%)	0.003
Stillbirth	0 (0%)	2 (6%)	0.09
Miscarriage	1 (2%)	2 (6%)	0.3
Apgar at 1 min (mean)	9.4	7.1	<0.0001
Apgar 5 min (mean)	9.4	7.8	<0.0001
Apgar at 10 min (mean)	9.8	8.3	0.001
Congenital infection	1 (2%)	6 (19%)	0.024

CPAP—continuous positive airway pressure; NICU—neonatal intensive care unit.

**Table 3:** Comparison of perinatal and maternal outcomes between elective cerclage and emergency cerclage groups.

	Elective (n = 43)	Emergency (n = 32)	*p*
Highest maternal WBC during hospitalization (mean), G/L	10.5 ± 3.2	12.2 ± 3.5	0.011
Highest maternal CRP during hospitalization (mean), mg/L	8.9 ± 12.3	10.7 ± 13.2	0.25
Pre-birth MgSO_4_	3 (7%)	7 (22%)	0.09
Gestational week at cerclage removal (mean)	36.1 ± 2.2	31.4 ± 5.6	<0.001
PPROM	6 (14%)	4 (13%)	0.416
Birth week (mean)	36.1 ± 3.88	31.4 ± 5.68	<0.001
Cesarean section	23 (53%)	19 (59%)	0.172
Pregnancy prolongation following cervical cerclage insertion (mean), weeks	18.6 ± 5.4	12.2 ± 6.4	<0.0001
Delivery before 28 weeks of gestation	2 (5%)	8 (25%)	<0.0001
Delivery between 28 and 32 weeks of gestation	6 (14%)	8 (25%)	0.192
Delivery between 33 and 38 weeks of gestation	15 (35%)	9 (28%)	0.246
Delivery after 38 weeks of gestation	20 (47%)	7 (22%)	0.041

PPROM—preterm premature rupture of membranes.

## Data Availability

Data are available from the corresponding author upon reasonable request.
